# Retraction: Circular RNA circ-0016068 promotes the growth, migration, and invasion of prostate cancer cells by regulating the miR-330-3p/BMI-1 axis as a competing endogenous RNA

**DOI:** 10.3389/fcell.2025.1640193

**Published:** 2025-06-13

**Authors:** Frontiers Editorial Office

The journal retracts the 26 August 2020 article cited above.

Following publication, concerns were raised regarding the integrity of the images in the published figures. The authors and their institution have remained unresponsive to multiple requests for the raw data for this study. Given extant concerns over the validity of the study, the article has been retracted.

This retraction was approved by the Chief Editors of Frontiers in Cell and Developmental Biology and the Chief Executive Editor of Frontiers. The authors have not responded to correspondence regarding this retraction.

